# Diphtheria and Its Management

**Published:** 1884-07

**Authors:** 


DIPHTHERIA AND ITS MANAGEMENT. By Thomas Nichol, M. D., LL. D., B. C. L. Pp. 22; price, 10 cents. Montreal: W. Drysdale & Co., 1884.

This is No. 1 of the *Montreal Tracts on Homœopathy*, popular tracts for the laity; and gives a succinct history of diphtheria and shows the greater success of Homœopathy in its treatment. It is well-written and should be the means of proving to the laity that there *is* an escape from the appalling statistics of allopathic treatment in this dread disease.

